# The Bacterium *Pantoea ananatis* Modifies Behavioral Responses to Sugar Solutions in Honeybees

**DOI:** 10.3390/insects11100692

**Published:** 2020-10-12

**Authors:** Ricarda Scheiner, Sina Strauß, Markus Thamm, Gerard Farré-Armengol, Robert R. Junker

**Affiliations:** 1 Behavioral Physiology & Sociobiology, Biocenter, Am Hubland, University of Würzburg, 97074 Würzburg, Germany; strauss.sina@web.de (S.S.); markus.thamm@uni-wuerzburg.de (M.T.); 2 Department of Biosciences, University of Salzburg, Hellbrunnerstraße 34, 5020 Salzburg, Austria; gerardfa1987@gmail.com (G.F.-A.); robert.junker@uni-marburg.de (R.R.J.); 3 Evolutionary Ecology of Plants, Department Biodiversity of Plants, Faculty of Biology, Philipps-University Marburg, Karl-von-Frisch-Str. 8, 35043 Marburg, Germany

**Keywords:** plant bacteria, bacterial spread, sucrose responsiveness, *Apis mellifera*

## Abstract

**Simple Summary:**

Honeybees are important pollinators, and may contribute to the spread of plant bacteria during their foraging trips. Some of these bacteria, such as *Pantoea ananatis*, can become deleterious to crops, leading to leaf blotches, die-back, bulb rot, and fruit rot. It is unknown whether honeybees sense the bacteria in the nectar of flowers. We aimed to determine if bees can perceive these bacteria in sugar solutions and if they are deterred by them. Our results indicate that honeybees can perceive these plant bacteria only in high concentrations, which deters them from drinking the respective sugar solution. They may therefore spread *P. ananatis* bacteria between flowers in field-realistic densities during foraging.

**Abstract:**

1. Honeybees, which are among the most important pollinators globally, do not only collect pollen and nectar during foraging but may also disperse diverse microbes. Some of these can be deleterious to agricultural crops and forest trees, such as the bacterium *Pantoea ananatis*, an emerging pathogen in some systems. *P. ananatis* infections can lead to leaf blotches, die-back, bulb rot, and fruit rot. 2. We isolated *P. ananatis* bacteria from flowers with the aim of determining whether honeybees can sense these bacteria and if the bacteria affect behavioral responses of the bees to sugar solutions. 3. Honeybees decreased their responsiveness to different sugar solutions when these contained high concentrations of *P. ananatis* but were not deterred by solutions from which bacteria had been removed. This suggests that their reduced responsiveness was due to the taste of bacteria and not to the depletion of sugar in the solution or bacteria metabolites. Intriguingly, the bees appeared not to taste ecologically relevant low concentrations of bacteria. 4. *Synthesis and applications.* Our data suggest that honeybees may introduce *P.* *ananatis* bacteria into nectar in field-realistic densities during foraging trips and may thus affect nectar quality and plant fitness.

## 1. Introduction

Many insects such as honeybees (*Apis mellifera*) are important pollinators globally. About 75% of major field crops, fruits, and vegetables produced for human consumption require insect pollination [1]. Similarly, the vast majority of wild plants depend on insect pollination [2,3]. Honeybees are among the most efficient pollinators, not least due to their large colony size. They collect nectar, pollen, and resin from diverse plants. During their visits to flower blossoms, bees frequently come in contact with microorganisms such as fungi and bacteria or viruses. It was therefore suggested that honeybees likely transfer plant pathogens such as bacteria from flower to flower during foraging and from the flowers to their hive [4,5]. Thus, they might contribute to the wide spread of fire blight, caused by the bacterium *Erwinia amylovora* [6,7], and other plant infections. Whether honeybees are capable of tasting bacteria in plant nectar, however, is completely unknown. A widely distributed bacterial family is the Enterobacteriaceae [8]. One of its members is *Pantoea ananatis*, a gram-negative facultatively anaerobic bacterium which was discovered in pineapple in 1928 [9]. P. ananatis was first described by Serrano (1928) as Erwinia ananas [9]. Mergaert et al. (1993) proposed the name Pantoea ananas [10], which was corrected to *Pantoea* ananatis by Trüper and De’Clari [11]. *P. ananatis* has induced plant diseases in 11 countries [12]. The bacterium is capable of infecting diverse hosts and living as an epiphyte. Hosts include maize and poplar trees, which are frequently visited by honeybees for their pollen [12,13,14]. *P. ananatis* can induce several disease symptoms including leaf blotches and spots [15], stalk rot [16], bulb rot and die-back [17], depending on the specific host.

However, *P. ananatis* is also associated with ice nuclease activity, which is used in biological pest control. Certain ice-nucleating strains of *P. ananatis* can considerably decrease the cold hardiness of mulberry pyralid larvae [18], suggesting a use of these bacteria in biological control of insect pests. Further, *P. ananatis* possesses antifungal and antibacterial activities, and can thus protect its host plants against infection by other pathogenic fungi and bacteria. The previously described strain of *Erwinia uredovora*, for example, was found to have in vitro antibacterial activity against *Erwinia amylovora*, causing fire blight [19]. Interestingly, *P. ananatis* has been reported to induce bacteremia in humans in rare cases [20].

One means of spreading bacteria such as *P. ananatis* might be through flower-visiting insects such as honeybees who could collect the bacteria from nectar and pollen during foraging [21,22]. An alternative means is through consumption of fruit juice of, for example, melons, which are infested with *P. ananatis* [23]. The extent to which honeybees contribute to spreading *P. ananatis* is currently unknown, but isolates from the closely related *Pantoea agglomerans* have been gathered not only from flowers but also from pollen loads, honey sacs, and freshly stored nectar in honeybee colonies [24]. Johnson et al. [25] noted that honeybees can show an avoidance to *Pseudomonas fluorescens* bacteria, which are used as control agent against *E. amylovora*, and Vanneste [26] observed that bees sometimes preened themselves after being dusted with pollen coated with this *Pseudomonas* strain, but not with pollen coated with *P. agglomerans*. Bacteria have also been shown to modulate sugar consumption in *Bombus terrestris*, [27] and yeast was demonstrated to modulate the sugar composition in nectars [28], which might affect the choice behavior of honeybees. Honeybees have a fine sense of taste and can differentiate very well between different concentrations of sugar in a solution when stimulated at their antennae [29,30], whereas the proboscis is less sensitive. In this study we aimed to determine if pollen and nectar foragers of the honeybee are able to sense *P. ananatis* bacteria in aqueous sugar solutions and if their responsiveness to sugar solutions is affected by high bacterial concentrations.

## 2. Materials and Methods

### 2.1. Solutions

We obtained the strain 26SR6 of *P. ananatis*, which was originally isolated from corn and grown on nutrient agar with 2.5% glycerol [31]. To produce bacteria glycerol stocks, 10 µl of the original *P. ananatis* stock solution was dissolved in 50 mL LB media (Luria/Miller, Carl-Roth, Germany) and incubated overnight at 37 °C on an orbital shaker. Afterwards, optical density at 600 nm (OD_600_) was determined three times. The mean of the three measurements was used for calculating the number of colony-forming units. Means of all solutions were about OD_600_ ~ 1.0 (minimum: OD_600_ = 0.933; maximum: OD_600_ = 1.073). After pelleting the bacteria (10 min, 4427.28× *g*) they were re-suspended in 0.5 Vol 60% (*w/v*) sugar solution (glucose, fructose, or sucrose) and filled with 0.5 Vol 60% (*v/v*) glycerol. Thus, the concentration of bacteria was 125 times higher than in the growth media with OD_600_ ~ 1.0 and the concentration of sugar was adjusted to 30% (*w/v*). The glycerol stocks were stored at −20 °C and were used to prepare the test solutions (see below).

### 2.2. Preparation of Honeybees

Returning honeybee foragers from two colonies with naturally mated queens were captured individually at the hive entrance in the morning and classified as pollen foragers (filled pollen baskets) or nectar foragers (without filled pollen baskets). The latter group may have comprised water collectors, which cannot be differentiated from nectar foragers by sight, but their number is usually very low (max. 5% [32]) and negligible. Bees performing orientation flights usually do not fly in the morning and can therefore also be excluded. Subsequently, bees were immobilized by cooling on ice and mounted in holders as described in [30]. Each bee was placed in a holder in a way that she could freely move her antennae and mouthparts. Behavioral experiments started one hour after mounting [30]. In the meantime, bees were maintained in a humid chamber at room temperature.

### 2.3. Influence of Bacteria and Their Soluble Metabolites on the Responses of Honeybees to Different Sugar Solutions

In this experiment we aimed to determine if different concentrations of *P. ananatis* in a sugar solution affect the proboscis extension responses (PERs) compared to pure sugar solution. We studied responses to antennal stimulation with 30% pure sucrose, fructose, and glucose solutions, and the same solutions containing different concentrations of *P. ananatis*. For these experiments we produced five 10-fold dilutions (D1–D5) with 30% (*w/v*) sugar solution (glucose, fructose, or sucrose) based on the glycerol stocks described above [27], with D1 containing the highest and D5 the lowest bacterial concentration. Although we cannot state the exact density of bacteria in each of the solutions, we estimated the density based on a corresponding growth solution with an OD_600_ ~ 1.0 of *Escheria coli* bacteria that contained approximately 8 × 10^8^ colony-forming units (cfu)/µl [33]. Corresponding test solutions were dilution 1 (D1): 10^7^ cfu/µl, dilution 2 (D2): 10^6^ cfu/µl, dilution 3 (D3): 10^5^ cfu/µl, dilution 4 (D4): 10^4^ cfu/µl, dilution 5 (D5): 10^3^ cfu/µl. To aid interpretation, we used these cfu/µl values in the figures and in Table 1. Statistical results are driven by the dilution factor and thus remain valid despite differences in the optical density between *E. coli* and *P. ananatis*.

The solutions were made fresh on each experimental day and cooled on ice. To test if honeybee foragers are capable of perceiving the bacteria in a less attractive, lower concentration of sugar solution, we stimulated the antennae of the bees either with 3% sucrose solution or with the same solution containing different concentrations of *P. ananatis*. Only honeybees showing 100% responsiveness to the pure sugar solutions with PER were selected for this experiment. The comparatively large number of individual tests could not affect satiation levels, because bees were only stimulated at their antennae and were not allowed to feed on the sugar solutions [34]. Pollen and nectar foragers were tested separately because they may respond differentially to the bacteria, given their different foraging experience. Having recovered from immobilization and mounting, each pollen and nectar forager was sequentially stimulated at the antennae with a pure sugar solution and with the same sugar solution containing an increasing concentration of *P. ananatis*. At each stimulation of the antennae with sugar solution, we recorded the occurrence of the proboscis extension response (PER). Tests were performed in the following order: 30% sugar, D5, 30% sugar, D4, 30% sugar, D3, 30% sugar, D2, 30% sugar, D1. The intertrial interval between the pure sugar solution and the sugar solution containing a certain concentration of *P. ananatis* bacteria was 2 min to avoid intrinsic sensitization [30].

In the following experiment, we aimed to determine if the reduced responsiveness to a highly concentrated sugar solution contaminated with a high concentration of bacteria resulted from the deterrence due to the taste of the bacteria or if the presence of bacteria caused changes in the properties of the solution. For instance, the solution could include the secretion of soluble metabolites or the reduction of the sugar concentration. For this experiment, we selected a 30% sucrose solution because this normally yields a high response rate of the honeybees [35,36]. We diluted serial concentrations (M1–M5, according to the description above and corresponding to D1–D5), stored them at room temperature for 30 min, and subsequently centrifuged the solutions at 3380.83× *g* for 5 min. This short and mild centrifugation step reduces the bacteria in the supernatant dramatically without destroying them. Centrifugation at 10,000× *g* was shown to reduce bacteria from the solution up to 99% [37], but forces greater than 5000× *g* have been shown to cause bacterial damage [38], which we wanted to avoid. Furthermore, smaller soluble molecules will not sediment during centrifugation because this needs substantially higher centrifugation speed and time. The upper part of the clear supernatant was then chosen for the behavioral tests. Similar to the previous experiment, we tested responses to the pure 30% sucrose solution and to increasing concentrations of *P. ananatis*-supernatant in 30% sucrose.

### 2.4. Do P. ananatis Bacteria affect Responsiveness Measured at the Proboscis and Consumption?

To quantify the responsiveness of pollen foraging bees and nectar foraging bees at the proboscis, which is usually less sensitive than the antenna [39], we stimulated each bee at her antenna with a 30% sucrose solution and observed if proboscis extension occurred. When the bee extended her proboscis, we presented 3 µl of a second sugar (pure or contaminated with *P. ananatis*) solution to her proboscis and noted whether the bee drank this solution. When the bee drank the entire 3 µl we recorded a positive response. If the bee only touched the sugar water droplet or did not drink the entire droplet, we recorded a negative response.

### 2.5. Determining Responsiveness to Increasing Sucrose Solutions with or without Bacteria

To determine the extent to which honeybees are capable of sensing a certain concentration of bacteria in increasing sucrose concentrations ranging from very low (0.1%) to high (30%) with their antennae, we produced increasing sucrose concentrations with and without bacteria. We first tested responses of all nectar and pollen foragers to stimulation of the antennae with the following sucrose concentrations applied in ascending order: 0.1%; 0.3%; 1%; 3%; 10%; 30% [30,32,36,39]. Then we calculated the sum of responses for each bee and divided the population of bees in two groups that were equal with respect to their sucrose responses, which constitutes their gustatory response score (GRS) [30]. One group was subsequently stimulated with increasing sucrose concentrations containing a bacteria concentration that corresponded to solution D2 in each sucrose concentration, while the second group with equal sucrose responsiveness in the pre-test was stimulated with increasing pure sucrose concentrations. Subsequently, we calculated the GRS again and compared it between the groups tested for pure sucrose solutions and those tested with sucrose solutions containing bacteria corresponding to solution D2.

### 2.6. Statistical Analysis

The experiments on responsiveness resulted in binary data in which the honeybees showed a proboscis extension response PER (1) or not (0). To test whether an increasing density of bacteria is associated with a reduced probability of honeybees to show a PER, we used a generalized linear models (GLM) with binomial error distribution and logit link function. We used the response probability (0 or 1) of the bees as the response variable and the assumed density of bacteria (cfu/µl in dilutions D1–D5), the honeybees’ task (nectar or pollen forager), and the interaction between density and task (density × task) as explanatory variables. This full model was reduced in a stepwise manner and finally compared to a neutral model containing no explanatory variables using an analysis of deviance [40]. If the two models were significantly different from each other, the explanatory variable that was excluded from the model was considered to have an effect on the responses of the honeybee. In addition to the GLMs, we estimated nonlinear least squares of the parameters a and b of logistic functions $r=\frac{e^{a+bd}}{1+e^{bd}}$, where *r* = proportion of honeybees displaying PER at a given density of bacteria in the solution and *d* = density of bacteria, describing binary responses and reflecting the logit link functions used for the GLMs [40]. For each experiment, we used the estimates of *a* and *b* and set *r* = 0.5 (i.e., half of the honeybees displayed PER and consumed the solution) to calculate the density of bacteria *d* that is required to prevent 50% of honeybees from consuming the solution. All of these statistical analyses were performed with the statistical computing software R (R version 3.6.2 (2019-12-12)—“Dark and Stormy Night”, R Core Team 2018). The gustatory response scores were compared between foragers tested with pure sucrose solutions and those tested with increasing sucrose concentrations containing *P. ananatis* using Mann–Whitney U tests because these scores did not follow normal distribution (SPSS 25, IBM).

## 3. Results

### 3.1. Responsiveness of Honeybees to Sugar Solutions Contaminated with Bacteria

**Figure 1:**
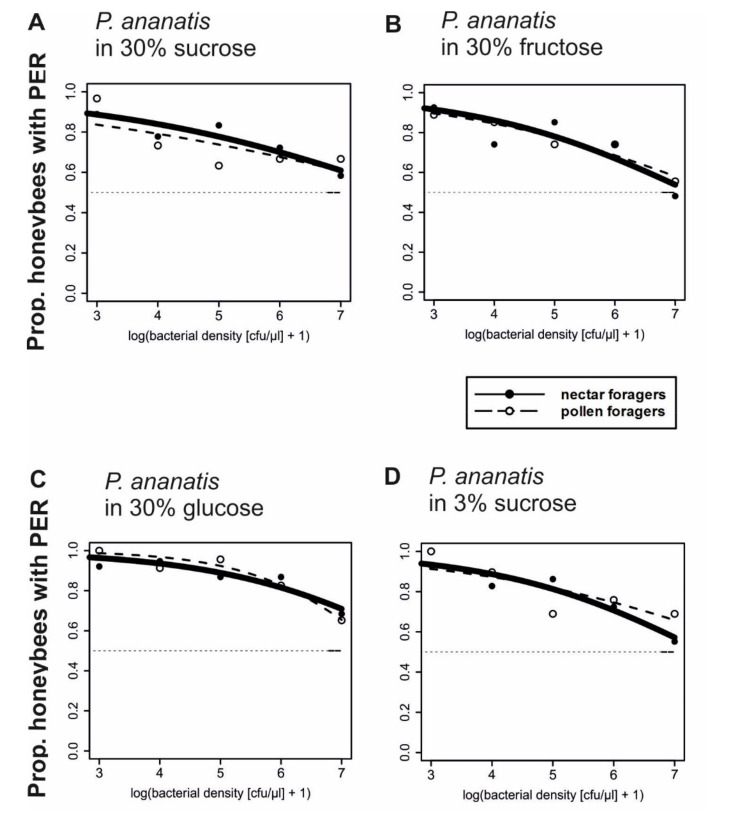
Proportion of honeybee nectar and pollen foragers accepting different aqueous sugar solutions containing variable densities of *P. ananatis* bacteria and responding with proboscis extension response (PER) to antennal stimulation. Regression lines represent the logistic functions on binary data of occurrence of PER: (**A**) 36 nectar foragers and 30 pollen foragers; (**B**) 27 nectar foragers and 27 pollen foragers; (**C**) 31 nectar foragers and 30 pollen foragers; (**D**) 29 nectar foragers and 29 pollen foragers.

We tested responsiveness of honeybee foragers to high (30%) and low (3%) sugar concentrations that were inoculated with different concentrations of *P. ananatis*. All solutions were applied to the antennal tips. By a significant margin, most bees showed PER at low concentrations of *P. ananatis* in a 30% sucrose solution (Figure 1A and Figure S1A), in a 30% fructose solution (Figure 1B and Figure S1B), in a 30% glucose solution (Figure 1C and Figure S1C), or in a 3% sucrose solution (Figure 1D and  Figur S1D  ). However, the probability for responding to the sugar solutions decreased significantly with increasing concentrations of bacteria, regardless of whether low (3%) or high (30%) sugar concentrations were contaminated (Table 1, Figure 1). This effect of bacterial density in the sugar solution on PER was observed in both pollen and nectar foraging bees (Table 1, Figure 1) to a similar extent. These experiments demonstrate that honeybees are deterred by high bacterial density of *P. ananatis* in a sugar solution, when they sense the bacteria with their antennae. This is regardless of whether these bees normally collect nectar or pollen.

**Figure 2:**
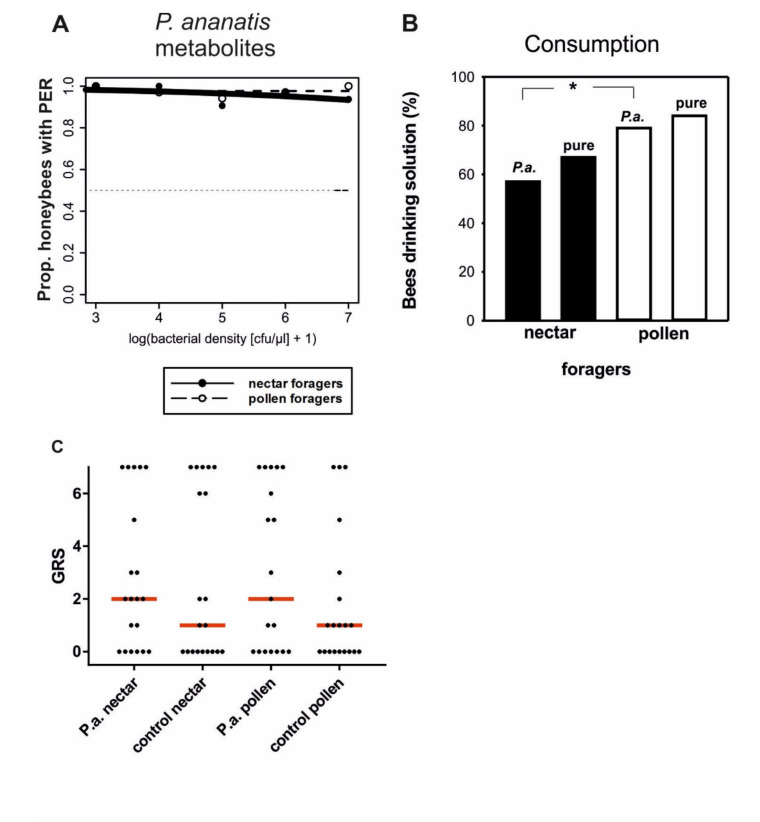
(**A**) Proportion of honeybee nectar and pollen foragers accepting 30% sucrose solution containing soluble metabolites (M1–M5) of *P. ananatis*. Regression line represents the logistic function on binary data of occurrence of proboscis extension response (PER). (**B**) Proportion of honeybee nectar and pollen foragers consuming 3 µl of pure sugar solutions vs. that of bees drinking the same sugar solution containing high concentration of *P. ananatis* (D1). Numbers of bees tested: P.a. in sucrose solution: 42 nectar and 43 pollen foragers; pure sucrose solution: 42 nectar and 43 pollen foragers. The significant difference in the proportion of pollen and nectar foragers drinking the solution containing *P. ananatis* is marked by an asterisk (*: *p* < 0.05). (**C**) Gustatory response scores (GRS) of nectar and pollen foragers that were stimulated at their antennae with either pure sucrose solution or a sucrose solution containing low densities of *P. ananatis* (corresponding to D4). Groups did not differ significantly from each other. Dots indicate individual bees. The red line shows the median GRS. Numbers of bees tested: nectar P.a. = 20; nectar control = 20; pollen P.a. = 19; pollen control = 20.

To determine if the deterring effect for honeybees results from the bacteria themselves, the bacteria’s secreted soluble metabolites, or the reduced sugar concentration of the solution, we removed the bacteria from the solutions and tested the responsiveness of the bees to antennal stimulation with that solution. When bacteria were removed from the sugar solution, honeybees responded similar to the case of the pure sucrose solution (Figure 2A, Table 1). Neither pollen foragers nor nectar foragers appeared to detect the presence of possible metabolites of *P. ananatis*.

As an additional measure of the effect of the bacteria on the responsiveness of the bees we calculated the density of bacteria that deterred 50% of the bees from responding. We found a broad range of bacterial densities that are required to prevent 50% of the bees from showing PER (Table 1). Intriguingly, neither the task of the bee nor sugar type had a significant effect on the bacterial densities required to prevent 50% of the bees from showing PER (ANOVA: *F*_1,14_ ≤ 2.76; *p* ≥ 0.119).

### 3.2. Probability for Drinking of Solutions

Nectar and pollen foragers did not have a higher probability of drinking pure sucrose solution compared to sucrose solution containing a high density of *P. ananatis* bacteria (nectar foragers: χ^2^ = 0.05, n = 42, *p* = 0.82; pollen foragers: χ^2^ = 0. 31, n = 43, *p* = 0.58), suggesting that both groups of foragers did not sense the bacteria with their proboscis (Figure 2B). Intriguingly, pollen foragers did not differ from nectar foragers in their probability of drinking the pure sucrose solution (χ^2^ =3.32, *p* > 0.05, n_nectar_ = 42, n_pollen_ = 43). However, pollen foragers were more likely to drink sucrose solutions contaminated with high densities of *P. ananatis* than nectar foragers (χ^2^ = 4.71, *p* = 0.03; Figure 2B). Because pollen foragers are generally more responsive to sugar solutions than nectar foragers [29,30,34,35], we wondered whether pollen foragers would also differ from nectar foragers in their responsiveness to sucrose solutions that were contaminated with bacteria.

### 3.3. Responsiveness to Increasing Sucrose Concentrations Contaminated with Bacteria

In this experiment, we analyzed responsiveness of pollen and nectar foragers to increasing concentrations of sucrose that contained *P. ananatis* according to solution D4 or were sterile. The gustatory response scores (GRS) indicate that both pollen and nectar foragers did not discriminate significantly between pure sucrose solutions and solutions containing a low concentration of bacteria (Figure 2C; nectar foragers: U = 179, n_control_ = 20; n*_P. ananatis_* = 20; *p* = 0.55; pollen foragers: U = 153, n_control_ = 20; n*_P. ananatis_* = 19; *p* = 0.29, Mann–Whitney U Test).

## 4. Discussion

*Pantoea ananatis* is a frequent bacterium on flowers and has become a pest for some crops [12,13,14,15,16,17]. The bacterium might be transferred between flowers and plants by pollinators such as the honeybee [41]. Honeybees likely consume these and other bacteria along with nectar and pollen because the microbiome associated with honeybee surfaces and guts, in addition to their stored food, is dominated by bacilli [42].

In this study we aimed to determine if honeybees perceive floral bacteria with their main taste organs, i.e., their antennae and proboscis, and if they avoid sugar solutions contaminated with bacteria. The bumble bee (*Bombus terrestris*), for example, was shown to perceive bacteria in floral nectar and to display a reduced feeding preference for higher concentrations of these bacteria in nectar, whereas low concentrations were generally tolerated [27]. A similar phenomenon has been reported for hummingbirds [43]. Effects of epiphytic bacteria on feeding behavior appears more widespread than assumed hitherto and has even been reported for slugs. *Arion vulgaris* slugs, for example, refused lettuce leaves when they were inoculated with bacteria [44].

Our experiments clearly show that honeybees are capable of perceiving *P. ananatis* bacteria when the bacteria are offered to their antennae in high concentrations in sugar solutions. Regardless of their experience as pollen or nectar foragers, the honeybees avoided sugar solutions contaminated with high (D1 and D2) concentrations of these microorganisms when they were applied to their antennae, and displayed a dose-dependent reduction in proboscis extension responses.

When we stimulated the antennae of the bees with increasing sucrose concentrations containing a comparatively low concentration of *P. ananatis* bacteria (D4), however, the bees did not discriminate between pure sugar solutions and solutions containing bacteria. These data suggest that honeybees are not deterred by low concentrations of *P. ananatis* bacteria in nectar and may thus spread the bacteria in low and field-realistic concentrations. Sugar solutions in higher concentrations than usually found in nectar may prevent the growth of bacteria [45], suggesting that bacteria remained viable in our sugar solutions. However, future experiments should also investigate the interactions between bacterial densities, bacterial viability as a function of sugar concentration, and pollinator behavior to fully evaluate the effect of bacteria on pollinators and pollination.

Intriguingly, when we stimulated their probosces with sugar water containing a high (D1) concentration of *P. ananatis* bacteria, bees did not avoid these solutions. This points to a lower sensitivity to bacteria at the proboscis, as has been shown for sugar [46] and which correlates with a lower number of taste receptors on the proboscis [36]. This situation, however, is rather artificial, because honeybees normally probe the nectar with their antennae when they decide whether they should collect it. When the concentration of bacteria is high, the bees do not extend their probosces and will likely not collect nectar from the source. They are therefore unlikely to consume pollen or nectar inoculated with a high density of bacteria compared to food containing a low quantity of bacteria and are unlikely to transfer large amounts of bacteria between plants.

The reduced probability of responding to the sugar solutions was not an effect of the duration of the experiments such that honeybees reduced the responses towards the sugar solution due to fatigue. This was evident because they readily responded to nearly all of the sterile sugar solutions that were offered after each sugar solution containing bacteria until the end of the experiment (Figure S1).

Little is known about naturally occurring densities of bacteria in nectar and pollen. Some of the bacterial densities that we used in our experiments likely exceeded those reported for nectar (3.1 × 10^4^, [22]). However, our results suggest that bacteria in natural or agricultural ecosystems may not strongly affect the foraging behavior of honeybees because bacterial density is normally low and will not be perceived by the bees. Bacterial applications for biological pest control, in which bacteria are applied in much higher concentrations [41], however, might deter the honeybees and possibly reduce pollination. Earlier studies showed that different bacteria can affect the foraging behavior of pollinators at different concentrations [27], indicating that our present results may be transferred to other bacterial strains. However, our experiments were performed with restrained honeybees in the laboratory. Future studies should further investigate choice behavior of free-flying honeybees between food sources infested with bacteria and those without.

## 5. Conclusions

Our data suggest that honeybees are capable of perceiving high concentrations of *P. ananatis* bacteria in sugar solutions with their antennae, which inhibit their proboscis extension response. Bees are therefore unlikely to drink from these solutions. Thus, a high infestation with bacteria, such as that occurring during biological pest control, might ultimately result in reduced pollination, whereas an ecologically relevant low concentration of bacteria is unlikely to be perceived by the bees’ antennae and will not affect their behavior. The transfer of low concentrations of bacteria by honeybees from flower to flower may ultimately change the bacterial community in the floral nectar and affect the composition of nectar by changing the sucrose–fructose balance, and the pH value of the nectar in the long run [43,47]. These results support our suggestion that the honeybee may possibly introduce bacteria into the nectar and thus may positively or negatively affect nectar quality and plant fitness.

How the bees perceive the bacteria remains an open question because it is unclear which kind of receptors of the honeybee are stimulated by bacteria and how this is represented at the neuronal level. It is rather surprising that the honeybee can perceive bacteria at all, given their small repertoire of gustatory receptors. Because most of the 10 gustatory receptors of the honeybee still await their functional characterization [48], more time is needed to understand how bacteria in floral nectar lead to signals that reach the brain. It is conceivable that the honeybee does not possess receptors specifically tuned to the perception of bacteria, but rather that the responses of sugar receptors of this important pollinator are inhibited by the presence of large quantities of bacteria.

## Figures and Tables

**Table 1:** Effect of bacterial density (D1 to D5) and honeybees’ task (nectar or pollen forager) on the probability of proboscis extension response (PER). For each experiment, the results of the generalized linear models are given, i.e., the effect of density and task and the interaction between both explanatory variables. Significant effects are highlighted in bold. Additionally, the estimated density of bacteria at r = 0.5, i.e., the density of bacteria that prevents 50% of honeybees from showing PER, is given.

			Density × Task			Task			Density			Density at r = 0.5	
Data	Solution	Treatment	df	Deviance	*p*	df	Deviance	*p*	df	Deviance	*p*	Nectar	Pollen
P.a. 30% sucrose	30 % sucrose	bacteria	327	−0.024	0.877	328	−0.352	0.553	329	−15.909	<0.001	1.28 × 10^8^	2.92 × 10^8^
P.a. 30% fructose	30% fructose	bacteria	267	−0.079	0.779	268	−0.021	0.884	269	−20.792	<0.001	1.89 × 10^7^	5.15 × 10^7^
P.a. 30% glucose	30% glucose	bacteria	302	−1.414	0.234	303	−0.089	0.765	304	−22.244	<0.001	3.13 × 10^8^	4.91 × 10^7^
P.a. 3% sucrose	3% sucrose	bacteria	287	−0.005	0.941	288	−0.367	0.545	289	24.251	<0.001	3.10 × 10^7^	3.53 × 10^8^
P.a. metabolites	cell suspension	no bacteria	327	−0.946	0.331	328	−0.552	0.458	329	−1.3074	0.253	7.41 × 10^14^	NA

## Supplementary Materials

The following are available online at https://www.mdpi.com/2075-4450/11/10/692/s1, Figure S1 Proportion of bees showing the proboscis extension response to pure sugar solution or sugar solution containing *P. ananatis* bacteria. Repeated stimulation of the antennae of honeybee foragers does not lead to fatigue, because responses to pure sugar solutions offered in between sugar solutions contaminated with different amounts of bacteria remained high until the end. Proportion of bees showing the proboscis extension response to pure sugar solution or sugar solution containing *P. ananatis* bacteria. Raw data corresponding to Figure 1.
