# Supplementary figures and images for: The Bacterium Pantoea ananatis Modifies Behavioral Responses to Sugar Solutions in Honeybees
Source: Insects. 2020 Oct 12;11(10):692. doi: 10.3390/insects11100692 (PMC7601739; doi:10.3390/insects11100692)

Figure S1

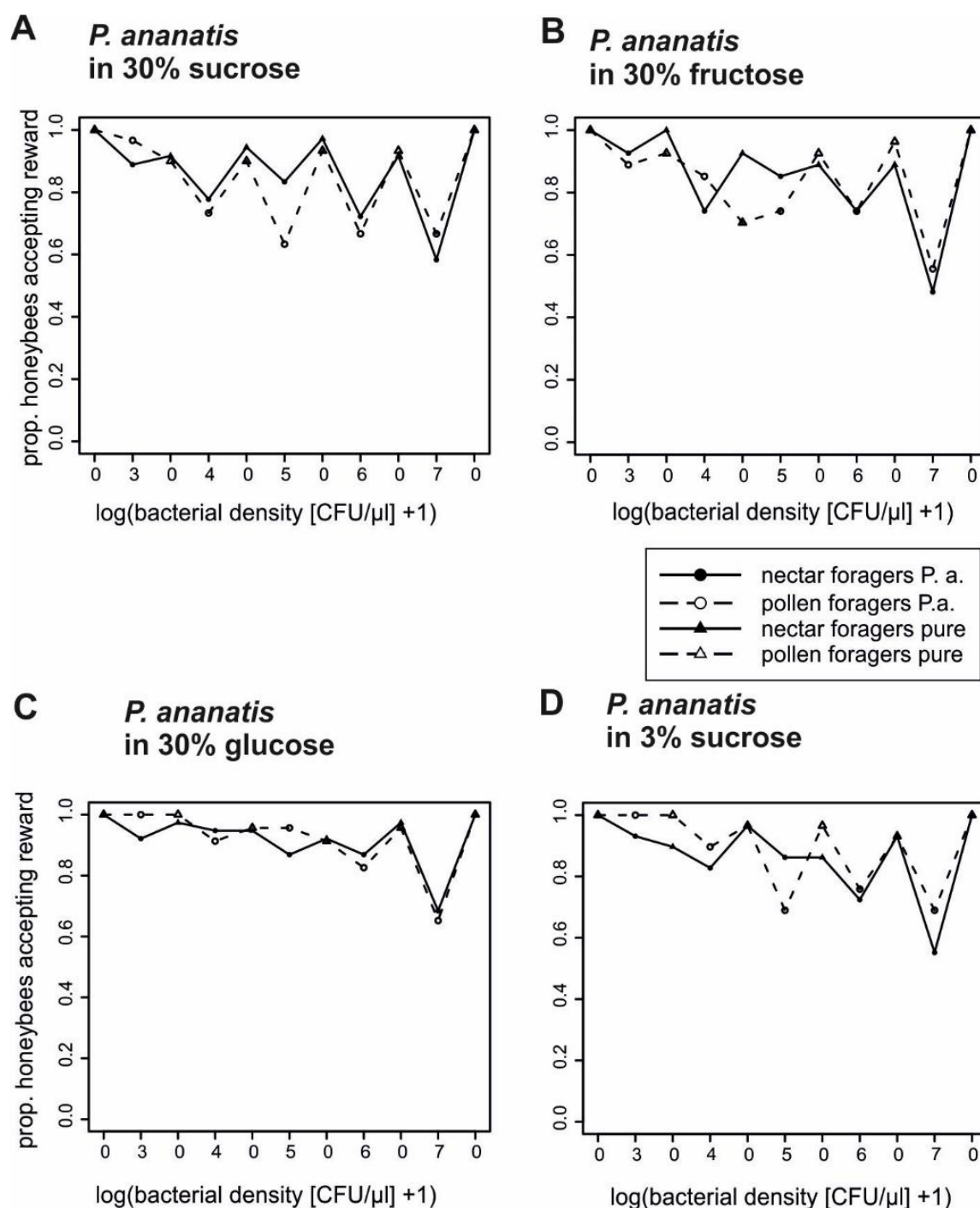

Supplement: Supplementary file 1 [file insects-11-00692-s001.pdf]
